# Contextualizing Evaluation in Research Consortia: A Reflective Case Study from the Research Centers in Minority Institutions (RCMIs) Program

**DOI:** 10.3390/ijerph23060747

**Published:** 2026-06-02

**Authors:** Kelly A. Laurila, Suzanne M. Randolph Cunningham, Lakesha Stevenson, Melissa Tarasenko, Lauren M. Ramsey, Carlamarie Noboa-Ramos, Katherine Matos, Akash Dania, Angela Sy

**Affiliations:** 1 Center for Community Health and Engaged Research, Northern Arizona University, Flagstaff, AZ 86011, USA; 2 Research & Evaluation, The MayaTech Corporation, Silver Spring, MD 20910, USA; 3 Center for Cancer Research and Therapeutic Development, Clark Atlanta University, Atlanta, GA 30314, USA; 4 HealthLINK Center for Transdisciplinary Health Research, San Diego State University Research Foundation, San Diego, CA 92182, USA; 5 Center for Community Prevention and Treatment Research, The MayaTech Corporation, Silver Spring, MD 20910, USA; 6 Grupo Nexos, Inc., Guaynabo, PR 00971, USA; 7 RCMI-Center for Collaborative Research in Health Disparities, University of Puerto Rico—Medical Science Campus, San Juan, PR 00936, USA; 8 The Interdisciplinary Health Research, Delaware State University, Dover, DE 19901, USA; 9 Department of Tropical Medicine, Medical Microbiology, and Pharmacology, John A. Burns School of Medicine, University of Hawaii, Honolulu, HI 96813, USA

**Keywords:** meta-evaluation, non-quantitative approaches, mixed methods, research capacity building (RCB), Research Centers in Minority Institutions (RCMIs), evaluation domains

## Abstract

**Highlights:**

**Public health relevance—How does this work relate to a public health issue?**

- By expanding consortium-wide evaluation to include non-quantitative approaches, institutions focused on building research capacity can generate meaningful, contextually grounded evidence needed to advance health disparities research.

**Public health significance—Why is this work of significance to public health?**

- Understanding how research capacity is built and evaluated determines the quality and relevance of public health science.
- Non-quantitative evaluation approaches provide explanatory insight into mechanisms of change within complex systems, strengthening the effectiveness and sustainability of health disparities research programs.

**Public health implications—What are the key implications or messages for practitioners, policymakers and/or researchers in public health?**

- Combining quantitative benchmarks with qualitative strategies provides a better understanding of program impact across consortium sites.
- This integrated approach helps evaluators refine programs and enables funding agencies to assess return on investment.

**Abstract:**

In 2020, evaluators within the Research Centers in Minority Institutions (RCMIs) program proposed a conceptual framework identifying four primary evaluation targets: scientific productivity, scientific collaboration, professional growth, and research resources. This study extends prior work by capturing the contextual and process-oriented dimensions of program impact. This reflective practice-based project examines how non-quantitative approaches complement traditional metrics to better characterize RCMI outcomes. Evaluators representing ten RCMI sites participated in a multi-site case study guided by three questions addressing: (1) qualitative evidence of impact beyond metrics; (2) challenges and successes in implementation of non-quantitative methods; and (3) potential expansion of evaluation targets. Evaluators provided descriptive responses, generating a 22-page dataset that was analyzed thematically. Thirteen non-quantitative evaluation domains emerged: investigator consultations, investigator productivity, investigator success, community partnerships, intra-RCMI collaborations, implementation of team science, career progression, programmatic support, mentoring support, impact on RCMI affiliates, intellectual resources, physical resources, and faculty hires. Key challenges included inconsistent data capture and limited evaluation resources, while successes highlighted improved cross-site learning and visibility of program impact. Findings support retaining the original evaluation targets while expanding the framework to include institutional transformation, equitable research environments, and longitudinal societal impact. A conceptual map was developed to depict how mixed methods that include non-quantitative approaches can yield RCMI evaluations that expand upon the current approach, which relies primarily on quantitative data. The authors recommend quantitative targets and non-quantitative strategies to provide context, communicate evidence of success, and inform programmatic changes to deepen the findings and strengthen the rigor of RCMI evaluation practices.

## 1. Introduction

### 1.1. Evaluating Research Capacity Building (RCB) and Impact

Research capacity building (RCB) centers aim to strengthen the ability of academic institutions to design, implement, and translate research into practical applications. The goal of RCB funding is to support multi-institutional, community-engaged consortia working to tackle complex health issues and reduce health disparities [1,2,3]. As these RCB programs evolve, evaluation has transitioned from tracking of scholarly products (e.g., grants, publications) to evaluation involving comprehensive and robust frameworks that measure research capacity, interest-holders’ engagement, and community impact [4]. Evaluation is critical for geographically dispersed, multi-partner research consortia that require strategies beyond simple outcome tracking [2,4,5].

According to the Centers for Disease Control and Prevention (CDC), evaluations should be relevant and utility-focused, providing actionable information that is in alignment with and responsive to interest-holders’ needs. Intentional planning is necessary at every stage: design, implementation and dissemination [6]. RCB evaluation provides a systematic examination of processes, inputs, and outcomes while considering institutional, economic, and geographic contexts [7,8,9]. Specifically, RCB evaluations aim to identify why centers succeed or face challenges with promoting learning and growth and to inform program modifications by tracking milestones and metrics [10].

Numerous evaluation frameworks and models have been developed to capture the process of how activities result in successes in translational research. The importance of linking process to outcomes in healthcare settings has been developed through an RCB evaluation framework for clinical settings with four levels of influence (individual, team, organizational, and network), as well as six principles for capacity building addressing these levels (building skills and confidence, developing linkages and partnerships, ensuring the research is “close to practice”, developing appropriate dissemination, investments in infrastructure, and building elements of sustainability and continuity). There is also a need for more RCB-focused studies that center on how to do evaluation in practice [11]. Understanding effective collaboration in RCB settings is critical. Thus, evaluating how investigators and decision makers (e.g., community partners or policymakers) work together is important, including identifying shared goals, balancing interest-holder values, building relationships and trust, allocating time to collaborate, and navigating organizational structures [10]. Boyd et al. call for more RCB-focused studies that center on how to do evaluation in practice [11].

### 1.2. Evaluation Frameworks

Evaluators of research centers may apply frameworks, especially when assessing the long-term impacts and benefits of research, including the Translational Science Benefits Model (TSBM), which focuses on clinical, community, economic and policy impacts [8,9,12]. Challenges associated with the TSBM include inconsistent definitions that impact data quality and missing elements of translational research training/workforce development [13]. Additional challenges include making direct associations between science and community impacts and translational lag time in moving research findings to community practice [14]. Emmons et al. recommend the expansion of the TSBM outcomes to better understand formative or process outcomes, such as implementation and capacity building, as antecedents to societal benefits by focusing on the processes through which impact efficiently and effectively happens [15]. While the TSBM is being used as a framework for research evaluations because it captures the societal benefits of research, the model lacks the granularity to explain how institutional capacity was built to achieve those benefits. While the TSBM measures the “what impact,” other evaluation and implementation science frameworks identify and depict the “how” and “why” of the organizational journey.

In contrast to the TSBM, which focuses on real-world benefits [14], the Consolidated Framework for Implementation Research (CFIR) centers on why implementation succeeds or fails. The complex interactions between an intervention’s characteristics, the outer setting of the organization, and the internal readiness of the individuals involved provide a framework for specific barriers and facilitators that occur during the process of change [16]. The RE-AIM (Reach, Effectiveness, Adoption, Implementation, and Maintenance) framework addresses the delays in translating scientific evidence into real-world practice and policy [17]. A Clinical Translation Science Award (CTSA) evaluation of community engagement applied RE-AIM for scalability and context-sensitivity of findings for the program [18]. RE-AIM incorporates outcomes for internal and external validity so that programs and policies are sustainable in clinical and community settings [19]. Developmental Evaluation provides a specific approach for complex, multi-partner initiatives that operate in dynamic environments. Developmental Evaluation supports real-time adaptation and decision-making within large-scale systems-change initiatives and alliances of multiple interest-holders beyond formative or summative evaluations [20]. Similar to Developmental Evaluation, Responsive Feedback is a framework for interventions that aim to collect timely data so that implementers can be flexible and adaptable to make program changes [21]. Developmental Evaluation and Responsive Feedback frameworks are well-supported by non-quantitative and mixed-methods approaches that enrich programmatic feedback to inform change.

Demonstrating research impacts on communities and society takes time [22]. While community reach (the breadth of community involvement) is relatively straightforward to evaluate, determining how research impacts those communities remains a challenge [23,24]. Recommendations for evaluating RCBs call for a mixed-methods approach that integrates various quantitative and qualitative data sources as a promising method to document impacts [25]. Luke et al. underscore the importance of approaches that document benefits to society but acknowledge that traditional efforts are mostly quantitative [14].

### 1.3. Consortium-Based Evaluation Approaches

Research centers often operate as part of a larger consortium of institutions, and the overall research consortium, vis-à-vis individual centers, is evaluated. Published RCB consortium evaluations (relevant to this project) focus on national consortia funded by the National Institutes of Health (NIH), intended to advance health solutions and expand institutional capacity for health disparities research. The first of these initiatives, the Transdisciplinary Collaborative Centers (TCCs), is focused on reducing health disparities through community-engaged partnerships that bring together researchers, community partners, clinicians, and policymakers to address complex, multilevel health inequities. Scarinci et al. outline a participatory, interest-holder-engaged evaluation framework designed to guide development, implementation, and assessment using triangulated mixed-methods approaches. Process evaluation strategies to establish a “blueprint” to ensure activities are implemented as planned should include questions about utilization of resources and satisfaction using activity logs; biannual surveys to inform partnership function and engagement; tracking dissemination activities (number of subscribers who opened newsletters intended for community partners); and annual progress reports to document progress toward aims [26]. Rollins et al. also underscore the importance of both qualitative and quantitative evaluation approaches and outline a number of primary and secondary data sources for RCB. Process and outcome evaluation questions should focus on capacity building, infrastructure development, research implementation strategies, and translational policy research activities [27]. While these studies [26,27] detail approaches at single TCC sites, they do not address suggestions for consortium-wide evaluation (engaging evaluators across sites in the consortium). These TCC evaluations do not address the labor intensiveness of participatory methods or the evaluation of long-term outcomes.

The second consortium, the CTSA, includes programs that are distinguished from the TCCs in that the CTSA is a national “collaborative consortium focused on bringing more treatments to all people more quickly through advancing clinical and translational science” [28]. The CTSA program sets standards for how it evaluates processes and outcomes, as well as evaluation benchmarks [4]. Evaluation domains include clinical research processes, careers, services used at the institution, economic returns, collaborations, and products. A number of metrics detail general descriptive approaches in combination with primarily quantitative measures. The CTSA program published a meta-evaluation documenting evaluation tools and techniques, evaluation challenges, and best practices across the CTSA program nationally [29]. Quantitative, qualitative, and mixed methods were used, but the study did not detail the use of these methodologies. Evaluation approaches were outlined, including the use of strategic planning tools such as logic and process models, milestones, formal evaluation plans, National Center for Advancing Translational Sciences (NCATS) common metrics, and scorecards. Hoyo et al. called for “rigorous, consistent, and well-coordinated, collaborative cross-hub evaluation processes” [29].

The third NIH-funded research consortium, the Research Centers in Minority Institutions (RCMIs), is the focus of this paper. The principal objective of the NIH-funded RCMI program is to expand national capacity for health sciences research to benefit communities [30]. The current RCMI program structure includes an Administrative Core, a Research Capacity Core (RCC), an Investigator Development Core with a Pilot Project Program (PPP), a Community Engagement Core (CEC), and an optional Recruitment Core, and research projects (RPs) funded through the RCMI program that receive support from all cores. Based on the program’s most recent Request for Proposals [30], the evaluation of the RCMI program assesses changes in institutional capacity and scientific impact in health disparities research by fostering collaborative basic, clinical, and behavioral studies; developing innovative research programs, tools, and technologies that yield high-impact, peer-reviewed outputs; and expanding biomedical research infrastructure, including cores, resources, and multidisciplinary capabilities through pilot projects, external funding, and strategic leveraging of institutional and NIH-supported programs. Although the program provides guidance on key evaluation domains, it is not overly prescriptive, allowing flexibility for site-specific approaches and priorities. At the same time, to enable meaningful cross-site assessments across the 23 funded institutions, it is essential that RCMI evaluators engage in shared learning to identify and advance best practices. By applying these principles of utility and intentional planning, RCMI evaluators have long sought to standardize impact reporting across the consortium, beginning with the establishment of a formal evaluation framework [5].

In 2020, RCMI evaluators created a conceptual framework and evaluation constructs to inform the development of potential common metrics for RCMIs [5]. Sy et al. identified four primary targets, including increase scientific productivity, increase scientific collaborations, foster professional growth, and expand research resources, and 12 secondary targets, including grants, peer-reviewed publications, scientific dissemination, community dissemination, pilot project specific productivity, research partners, community partners, early career investigators, underrepresented investigators, physical infrastructure, intellectual resources, and faculty hires [5]. The secondary targets outlined in that paper are quantitative in nature, with all examples provided being numeric (number of publications, increase in grants awarded, etc.).

The RCMI Coordinating Center (RCMI-CC), a consortium-level program, developed a logic model with four primary inputs, including financial, infrastructure, human, and knowledge resources. Outcomes focused on three levels, including individual investigators, RCMI Center level (individual RCMI sites), and the RCMI consortium, while impacts included population (disparity focus), economic, and policy metrics [31]. The article did not detail evaluation approaches. The RCMI-CC used secondary validated data sources to communicate the national public health impact of the RCMI program across 23 sites [32]. This included quantitative measures such as NIH grants awarded and publications using NIH RePORTER (an electronic tool that allows users to search a repository of NIH-funded grants) to access publications and patents resulting from NIH funding. Investigator profile data and patent records (obtained from the United States Patent and Trademark Office) were summarized using descriptive statistics. Qualitative (non-quantitative) domains were limited; descriptions of research conducted across the RCMI consortium were developed using word clouds based on top search terms in publications, program goals, and research project titles from “Centers-at-a-Glance” documents provided annually to the RCMI-CC. Content analysis was applied to communicate community impact using a monograph authored by members of the Community Engagement Core [33]. These evaluation studies used only descriptive non-quantitative approaches, which do not address formative or process outcomes to understand how implementation and capacity building occur [15].

Across these consortia, evaluation frameworks and approaches are limited in how well they guide the contextualization of the real-world data evaluators collect across complex, multi-partner health equity settings. Moreover, the gaps in current consortium-level RCB evaluation frameworks can lead to uneven implementation, evaluation fatigue, and difficulty demonstrating impact, especially in centers with limited resources or complex community histories.

While the three NIH-supported consortium models (TCC, CTSA, and RCMI) detailed above share a common goal of strengthening collaborative research capacity and translational impact, they differ substantially in evaluation orientation, implementation structure, and methodological emphasis. CTSA evaluations generally emphasize standardized benchmarking, shared metrics, and operational accountability across institutions. In contrast, TCC evaluations prioritize community-engaged and participatory approaches, focusing more heavily on partnership dynamics and implementation fidelity. RCMI programs integrate health disparities research, investigator development, and institutional capacity building across highly diverse institutional and community settings, requiring evaluation approaches that extend beyond conventional productivity indicators.

Although quantitative metrics such as grants, publications, partnerships, and infrastructure development remain important across all three models, limited guidance exists on how evaluators can systematically capture institutional transformation, collaborative processes, implementation experiences, and community impact across multi-site environments. Collectively, these models reveal a broader methodological gap in research capacity-building evaluation. Existing consortium-wide assessments largely emphasize quantitative outputs and standardized reporting while paying limited attention to reflective and contextualized approaches that explain how and why institutional and investigator capacity evolves over time.

### 1.4. Common Data Elements (CDEs) and Common Metrics

To facilitate comparative analysis across diverse research centers, there is a movement toward standardized common data elements (CDEs) in evaluation. CDEs typically measure four areas: “scientific productivity” evaluates grants submitted and awarded and publications [34,35,36]; “workforce development” measures outcomes to increase the biomedical research workforce through investigator collaborations and training; “infrastructure” assesses the availability and use of research programs and equipment necessary for research; and “community engagement” evaluates community involvement and participation in research [2]. Research center consortia have introduced evaluation data harmonization beyond the evaluation of single research center sites to CDE evaluation initiatives that enhance evaluation practice and reproducibility across multiple research centers in a consortium [1,37]. In NIH-funded multi-site research programs, adoption of shared measures enhances reporting quality, strengthens comparative analyses, and supports national-level program evaluation [1]. Ultimately, this alignment with common national evaluation data elements ensures that consistent elements (metrics and measures) across research institutions are captured in aggregate rather than fragmented, individual institutional metrics of research centers alone [2,32].

Specific CDEs for RCB consortia have been identified. For example, the RCMI program described two of the 13 CDEs focused on the Community Engagement Core (CEC): number, type (formal/informal), and duration (number of years) of “academic–community partnerships” (number of formal/informal partnerships) and “community-engaged research partnerships” (number of community partners) [33]. The CTSA program, which launched a common metric program in 2015, introduced three quantitative CDEs: median IRB review duration (research process), retention of scholars and trainees in the clinical and translation research field (career development), and publications and grant awards obtained by pilot awardees (scientific productivity) [14]. A recent scoping review identified eight (8) research capacity evaluation domains (bibliometrics, impact, ongoing research, collaboration activities, funding, personnel, education/academics, and recognition) with 42 quantitative metrics [38]. In a global health research capacity context, common metrics are also entirely quantitative, including the number of clinical trials, research activities, and publications [39]. Common metrics and CDE approaches are highly quantitative, missing the contextual information that conveys consortium-level and individual-site impact.

### 1.5. Purpose of This Paper

Despite recent RCB evaluation initiatives such as CDEs, the existing literature lacks non-quantitative (including qualitative or mixed methods) multi-site evaluation approaches for consortium-level programs [4,5,14,23,33,37,40]. While RCB evaluation is complex, consortium-wide RCB evaluations of multiple centers require multiple approaches. More robust (mixed-methods) RCB evaluations focused on building institutional and investigator health disparities research capacity (the core goals of the RCMI) are needed [32]. Strategies for assessing investigator development should include psychosocial factors such as “scientific identity” and “belonging” [41]. Qualitative success stories of RCMIs provide RCB contextual factors to describe the mechanisms of change [32]. Mixed-methods evaluations offer “explanatory” evidence, wherein qualitative and quantitative approaches are intentionally combined [42]. Furthermore, qualitative data can illuminate the processes underlying quantitative indicators [43]. Mixed methods help deepen our understanding of health disparities and identify solutions to health challenges by determining the contexts that explain “how” and “why” in complex settings [44].

The intention of this paper is to (1) characterize the non-quantitative evaluation approaches currently being implemented within RCMIs, (2) contextualize the successes and deepen our understanding of challenges RCMI evaluators face in implementing such evaluative approaches, and (3) utilize this information to inform an expanded evaluation model that incorporates mixed methods for documenting RCMI impacts on health disparities research capacity.

## 2. Materials and Methods

### 2.1. Methodological Approach and Theoretical Framework

Evaluators can use reflective practice to “recognize opportunities to humanize quantitative data to enhance its use” [45] and engage with other evaluators to reflect on evaluation questions, strategies for data collection, and sharing practices to inform next steps [46]. Meta-evaluation refers to the use of evaluation methods to monitor and examine an evaluation process for the purpose of enhancing its quality [4,40]. The goal of meta-evaluation is to uphold program evaluation standards, assessing an evaluation’s utility, feasibility, validity, and accuracy [47,48]. Ultimately, identifying evaluation challenges, as well as examples of successful implementation of evaluation methods and approaches, can provide evaluators with lessons learned to refine their practices and maintain effective assessment, monitoring and tracking systems [40,47]. In this paper, RCMI evaluators use meta-evaluation to reflect on evaluation practices at several sites and identify key domains for informing a more robust RCMI evaluation model. Contrary to many meta-evaluations that typically focus on assessing evaluation results to validate findings [4,40,49], the “evaluand” (that which is being evaluated) [49] for this project consists of consortium-wide approaches to expanding the existing RCMI evaluation domains [5]. Rather than examining the strengths and weaknesses of a program’s evaluation, the authors explored challenges and assets related to employing non-quantitative evaluation approaches.

The foundational work of Sy et al. [5] identified and described several quantitative RCMI evaluation domains. This paper presents a multi-site case study using practice-based examples that draw on the experiences of RCMI evaluators who implement non-quantitative evaluation approaches at their respective sites. A multi-site case study approach enables cross-site synthesis to identify common practices across sites [50]. The goal is to design a more robust evaluation model to contextualize the implementation, results, and impact of RCMIs in alignment with the primary targets identified by Sy et al. [5] and to characterize additional targets to consider when expanding the scope to focus on non-quantitative evaluation approaches.

### 2.2. Definitions

The authors chose “non-quantitative” rather than “qualitative” language in this paper to ensure a robust scope. The term qualitative might lead readers to assume the focus is limited to traditional qualitative methodologies such as interviews, ethnography, and focus groups. In addition to traditional qualitative approaches, non-quantitative methods include document/desk reviews using content analysis (meeting notes, technical reports, programmatic documents, including progress reports, abstracts, etc.), and open-ended survey questions. Additionally, these non-quantitative approaches include visual methodologies such as word clouds or Photovoice (a participatory action research and evaluation methodology that empowers participants to document their lived experiences through photography and accompanying narratives).

### 2.3. Project Development

RCMI programs utilize experts from multiple disciplines and resources to conduct site evaluations in accordance with the National Institute on Minority Health and Health Disparities (NIMHD) guidelines. Some sites include externally or internally positioned evaluators, while other sites rely on coordinators, program managers or core leads to perform evaluation activities according to the RCMI’s needs.

An informal Community of Practice (CoP) consisting of colleagues who serve as evaluators or coordinators (representing 10 of the 23 currently funded RCMI grant programs) started to convene monthly in the spring of 2025. Some individuals support evaluation for more than one RCMI. The CoP was drawn together with a common interest in sharing lessons learned and best practices for RCMI evaluation. This paper draws on the knowledge of RCMI sites with a wide range of tenures, including those in their first 5-year funding cycle and several with longstanding funding (e.g., 35 to 40 years of funding). The average duration of funding for the RCMIs contributing to this paper is 22 years.

### 2.4. Reflecting on Evaluation Practice: Guiding Questions

Through informal discussions, CoP members shared an interest in building on the previous work of RCMI evaluators [5] centered on primary evaluation targets (scientific productivity, scientific collaborations, professional growth, and research resources). The goal was to develop a broader understanding of non-quantitative evaluation strategies to assess RCMI impact. The CoP developed a set of three open-ended (qualitative) *guiding questions* informed by the team members’ experiences of being involved (directly or indirectly) in the evaluation of the RCMI program. CoP members were invited by email to contribute examples of non-quantitative approaches they employ in their RCMI work using a shared Google document (see the Supplementary Materials). Institution information was stripped from the analysis process as the aim of this paper is to disseminate lessons learned collectively, not to compare site-based evaluation practices or approaches. The three guiding questions were:

(1) What evaluation questions or lessons learned document or describe RCMI impact or outcomes beyond quantitative metrics and measures?
(2) Share any challenges and/or successes related (directly) to the evaluation work you described in the first question. Define success for your respective site based on the evaluation approaches you discussed in question 1 (not the results/findings from that evaluation work).
(3) Are there any missing primary targets that should be added (building on the Sy et al. 2020 paper [5])? What non-quantitative evaluation outcomes would you recommend for each new item you proposed?

Each question included four prompts to address the question with each of the four primary targets [5] in mind. For example, respondents were first asked to answer the question: *what evaluation questions or lessons learned document/describe RCMI impact or outcomes beyond quantitative metrics and measures?* Respondents were then prompted to address the question with specific approaches focused on evaluating scientific productivity, scientific collaborations, professional growth, and research resources. The intention was to ground our work in the high-level (primary) evaluation targets identified by Sy et al. [5].

Data collection lasted three months (19 September 2025 through 18 December 2025), including monthly reminders by email. The initial deadline was in October 2025, but the nature of the open-ended qualitative questions made it time-consuming to thoughtfully contribute. The extended deadline also took into consideration the federal government shutdown (1 October–12 November 2025). The group agreed to extend the deadline to ensure rich data collection.

The intention of this practice-based project was to engage the members of the CoP, not all 23 funded RCMI sites; thus, the sampling frame for this multi-site case study represents nearly half of the funded RCMI sites. Using a convenience sample, all members of the CoP (11) were invited by email to contribute; 72% (8 individuals) responded to the guiding questions. One individual opted out as their role in the evaluation was minimal. Two additional sites involved in the CoP planned to participate in this project but later opted out due to the large administrative and staffing burdens caused by federal grant terminations and reinstatements. It is important to note that three (3) of the 23 RCMI sites had their funding terminated and reinstated during the year this project was implemented (based on changes in federal funding priorities). Finally, not all RCMI sites have formal evaluators; the exact number of sites without formal evaluators is not well documented.

Respondents were asked to review each guiding question and share any best practices and valuable approaches from their respective RCMI evaluation processes. There was no expectation to provide a response to each item, noting that some newer sites may not yet have learned lessons to share. We used a Google document to allow for easy collaboration as the contributors are situated across sites. Individuals were encouraged to review others’ contributions to help them think of relevant approaches at their site and add breadth to the responses. They were also asked to stay focused on the question posed in their response. Respondents were directed to reflect on each question, thinking about the four (4) primary targets (scientific productivity, scientific collaborations, professional growth, and research resources) identified by Sy et al. [5].

### 2.5. Analysis

Once everyone submitted their contributions to the three open-ended questions, the team had a 22-page (single-spaced), text-based qualitative dataset. Five individuals, all of whom were RCMI evaluators, completed the initial review of the responses and agreed to a thematic analysis driven by the content presented in each question individually. To ensure data integrity and allow for consensus-building, the team used Excel spreadsheets to manage the analysis process for each of the three questions.

This reflective practice-based project (not a research study) is intended to inform more comprehensive and standardized evaluation for the RCMI consortia. The authors worked to ensure integrity in the analysis process by considering *credibility* (long-term engagement), *triangulation* of multiple data sources to verify findings, and *audit trails* to document project decisions and approaches to analysis [51]. In terms of credibility, the findings represent practice-based knowledge from evaluators serving RCMI sites across the geographic reach of the program, including insider and outsider perspectives (internal and external evaluators), and evaluators representing diverse demographic and disciplinary backgrounds, different lived experiences, and individuals who are early and late in their careers. As a reflective practice-based project, the team used one data source (open-ended responses to a set of guiding questions). The Google document used for data collection allowed the authors to consider others’ responses to elicit deeper reflection on their own RCMI site(s), non-quantitative evaluation practices, and provided the opportunity for informal consensus-building. The authors recognized that this approach could potentially contaminate the responses and introduce conformity bias, but nevertheless, they prioritized the potential benefit of utilizing the information contained within the document to prompt additional responses from evaluators. The responses provided were sufficiently detailed and contextually grounded to support a comprehensive analysis, offering depth and clarity that allowed key themes and insights to emerge. Audit trails included meeting notes and e-mails used to document decisions about the analysis process, as well as any changes that occurred.

Question 1: *What evaluation questions or lessons learned document/describe RCMI impact or outcomes beyond quantitative metrics and measures?*

The qualitative responses to question 1 were broken into 81 separate text excerpts in Excel that were thematically classified. Each respondent provided multiple strategies in their responses as they related to each primary target. Each strategy, for example, was analyzed as a unique text excerpt. In total, 73 text excerpts were included in the thematic analysis. Four items (4) were excluded because the item reported was quantitative, because the example item was too vague to classify, or because it was a planned strategy that had not been implemented. This paper focuses on evaluation approaches currently in practice. Additionally, four items (4) were reclassified as examples of a new primary target and were thus analyzed in relation to question 3.

Respondents were asked to address question 1 with evaluation questions or lessons learned that relate to each primary target (scientific productivity, scientific collaborations, professional growth, and research resources). As the analysis team split specific examples from large paragraphs into smaller text excerpts, it became clear that the primary target under which the larger paragraph was submitted did not apply to every example within the response. In total, 27 primary targets (of the 73 text excerpts) were reclassified. The analysis team used a data immersion process of reading through all the findings related to the primary target to identify emergent themes. The analysis approach was iterative to allow for consensus-building. Two team members were involved in coding the responses for question 1. Emergent themes (codes) were identified under each primary target by the analysis team for question 1 using an inductive approach. Using the theme categories, one team member conducted the initial coding. A second team member reviewed the themes (codes) and identified 14 cases where they felt a different theme should be applied and provided justification. Consensus-building was used to review cases where there was a disagreement and come to an agreement. This process was documented in Excel, where the reviewer agreed with the coding as is, agreed and proposed another possible code, or disagreed and suggested an alternative code. In cases where there was disagreement, the coders discussed their perspectives and came to an agreement. Subthemes were synthesized in tables (one per primary target).

Question 2: *Share any challenges and/or successes related (directly) to the evaluation work you described in the first question. Define success for your respective site based on the evaluation approaches you discussed in question 1.*

The goal of the analysis for question 2 was to compile challenges and successes reported by evaluators across all of the primary targets (not by individual target) using two questions focused on challenges and sharing successes. The raw data for this analysis were the verbatim responses in the overall dataset that pertained to question 2. However, verbatim sentences or entire passages of texts submitted for each strategy were only transferred to the question 2 Excel file if the response was retained in the question 1 dataset (i.e., strategies coded as “excluded” in the question 1 analysis were not retained for the question 2 analysis). The text included in the analysis was retained if it related to a “challenge” or a “success” in implementing the (non-quantitative) evaluation strategy, which resulted in a total of 26 excerpts. Two coders separately reviewed each text excerpt and came to a consensus on whether the data reflected a “challenge” or a “success.” The content analysis was conducted across all strategies, regardless of primary target. A first pass through the data focused on identifying and analyzing the “challenges” across all strategies (13 excerpts), and the second pass focused on the “successes” across all strategies (13 excerpts). Themes were generated through a structured review of all responses, and a consensus was reached by the two examiners assigned to question 2. A third pass of the entire question 2 dataset was conducted using generative AI to review the responses by strategy. Consistent with evolving qualitative research practices, AI was used as a tool to aid human-led verification and selection of quotes that fit the themes identified through interpretive decision-making, not as a replacement for qualitative or interpretive judgment. The themes generated were parallel to those identified manually. Finally, using generative AI, quotes were selected for specific “challenges” and “successes” to illustrate a theme. Two team members engaged in a process to review and validate the quotes to ensure they aligned with the reported challenges and successes. Like other accepted forms of computer-assisted qualitative analysis (e.g., Dedoose), AI functioned as a filter to accelerate the search for verbatim passages from the large dataset. Prompts included: “pull at least two one-sentence quotes directly from the evaluator narratives that illustrate each theme in the attached table”; “provide the accurate, verbatim quote in a third column in the table”; and “do not paraphrase or analyze.” Several safeguards ensured analytic rigor and trustworthiness. We constrained prompts to limit AI to retrieval rather than interpretation. Two researchers used verification against the original narratives to ensure accuracy and contextual fit of the quotes (e.g., that the passages before and after the quote supported our interpretation that the quote fit the theme). Prompts were archived as audit trails and potential use in replication studies, as is done in traditional qualitative analysis. The teams’ interpretive review determined which excerpts were included in the final analysis (i.e., theme development, meaning making, and final quote selection remained human). The researchers reviewed the quotes, and through consensus between two researchers, the final set of quotes for each theme was selected; thus, interpretive authority remained with the researchers, not AI.

Question 3: *Are there any missing primary or secondary targets that should be added? What qualitative (non-quantitative) evaluation outcomes would you recommend for each new item you proposed?*

The Excel data were sorted by the primary targets (again, respondents placed relevant responses under each primary target), so, for example, all items related to “scientific collaborations” were together in the spreadsheet. The analysis of question 3 was completed by two authors and followed a systematic content review of qualitative data provided by RCMI sites. The raw data consisted of 18 separate text excerpts, which were sorted according to the four original primary targets established by Sy et al. [5]. This iterative process was designed to determine if each excerpt represented a refinement of an existing target or the emergence of a new primary target. Consensus was reached using the same strategies described above. All 18 excerpts were included in the final synthesis because they provided substantive recommendations for the expansion of evaluation domains or identified non-quantitative outcomes currently in practice across the sites.

## 3. Results

### 3.1. Identifying and Contextualizing Non-Quantitative RCMI Evaluation Approaches (Guiding Question 1)

The first guiding question asked respondents to share “evaluation questions or lessons learned to document/describe RCMI impact or outcomes beyond quantitative metrics and measures.” This section focuses on the emergent non-quantitative evaluation approaches related to each of the primary targets [5] and provides concrete examples of approaches being employed across multiple RCMIs. Analysis of the 73 excerpts revealed that non-quantitative approaches were most used to evaluate professional growth (41%), followed by scientific collaborations (33%), research resources (18%), and, to a lesser extent, scientific productivity (8%).

#### 3.1.1. Scientific Productivity (Primary Target 1)

Within the primary target focused on scientific productivity, the key non-quantitative themes that emerged related to evaluation approaches that deepen understanding of how RCMI programming (investigator consultations) influences or inhibits progress (investigator productivity) and leads to deliverables (investigator success) (Table 1). The most salient themes were investigator productivity and investigator success, which were reflected in 67% of the scientific productivity text excerpts. Although there were commonalities with the previously identified secondary targets (e.g., grants, dissemination, publications) [5], the non-quantitative evaluation domains related to scientific productivity on facilitators and barriers to productivity and understanding “how” and “why” investigators are successful [44]. These evaluation domains move beyond a “blueprint” to ensure activities are implemented as planned [26] and gain insight into how RCMIs support community-focused research.

#### 3.1.2. Scientific Collaborations (Primary Target 2)

Within the primary target focused on scientific collaborations, the key themes centered on evaluating community partnerships, intra-RCMI collaborations, and implementation of team science. Approaches demonstrate the breadth of non-quantitative evaluation methods (Table 2). The most salient theme was community partnerships, which was reflected in 54% of the scientific collaboration text excerpts. These findings align with the work of Oortwijn et al., who underscore the importance of understanding collaboration (e.g., shared goals and relationships) among investigators and decision makers (e.g., community partners or policymakers) [10].

#### 3.1.3. Professional Growth (Primary Target 3)

Within the primary target focused on professional growth, the key themes centered on evaluating career progression, programmatic support, mentoring support, and the impact of RCMIs on investigators using an array of non-quantitative approaches for evaluation (Table 3). Career progression and programmatic support were the two most salient themes; both were reflected in 30% of the excerpts. Traditional qualitative evaluation approaches, such as interviews or open-ended survey questions, that evaluate professional growth were reported as ways to understand the impact of the RCMI on investigators and mentoring, as well as programmatic supports. Our findings indicate that RCMI evaluation has a strong focus on professional growth, which could inform what Sperling et al. describe as one of the missing evaluation elements of translational research training/workforce development in the TSBM model [13].

#### 3.1.4. Research Resources (Primary Target 4)

Within the primary target focused on research resources, the key themes include intellectual resources, physical resources, and faculty hires (Table 4). These themes align directly with the secondary targets described by Sy et al. [5]. However, the non-quantitative evaluation approaches identified in this project are notably different from the quantitative metrics described by Sy et al. [5], as the non-quantitative approaches focus on awareness/utilization, perceived changes in capacity, influence of consultations on analytic rigor, support needs and gaps, and contextualizing infrastructure enhancements. The most salient theme was intellectual resources, which was reflected in 54% of the text excerpts for this target area.

### 3.2. Utilization of Non-Quantitative Approaches: Challenges and Successes (Guiding Question 2)

The second guiding question asked respondents to share any challenges and/or successes related (directly) to the utilization of the evaluation strategy or approach described in the first question. The analysis for question 2 was conducted across strategies, not separately for each primary target. The 26 text excerpts analyzed for question 2 were equally reflective of successes (*n* = 13 excerpts) and challenges (*n* = 13 excerpts). Below, Table 5 summarizes the cross-cutting themes identified by evaluators as “successes” in implementing non-quantitative approaches, while Table 6 summarizes the cross-cutting themes identified by evaluators as “challenges” in implementing non-quantitative approaches. Each table is followed by a summary of the findings for each theme within the categories, along with illustrative quotes.

#### 3.2.1. Successes

Sites described notable successes in utilizing non-quantitative approaches to evaluation (Table 5). Following the table, specific findings are presented for each theme.

**Table 5:** Summary of themes identified as “successes” of implementing non-quantitative approaches to RCMI evaluations.

Successes (Themes)	Examples
*Strengthening Evaluation Infrastructure Centralized Systems*	Improved accuracy, consistency, and efficiency; better tracking of service requests and resource use
*Mixed-Methods and Narrative Approaches*	Combining quantitative and non-quantitative data deepens interpretation; exit and follow-up interviews provide contextualized insights into individual, institutional and community impacts
*Systematic Tracking of Research Productivity and Collaboration*	Coding publications and grants beyond tallies (i.e., for research foci) provides more descriptive impact; longitudinal tracking of impact is possible; selected strategies reveal collaboration patterns not just that collaborations exist
*Documenting Community-Engaged Collaborations*	Mixed-methods tools capture partnership development and community capacity-building outcomes
*Data-Driven Program Improvements*	Responses from surveys with open-ended items, interviews, and narratives directly inform program changes and strategic decisions
*Structured Use of IDPs and Mentorship Evaluations*	Systematic tools provide insight into professional growth and mentoring quality

##### Strengthening Evaluation Infrastructure Through Centralized Data Systems and Tracking Tools

The use of centralized data systems enhanced the accuracy, consistency, and efficiency of evaluation activities. Programs leveraged institutional databases, federal data sources, and electronic data capture platforms such as REDCap to streamline data collection and reduce reporting burden. Through various non-quantitative approaches, these systems supported the tracking of service requests, user characteristics, and resource utilization while also enabling evaluators to identify infrastructure needs through open-ended survey responses. The non-quantitative approach yielded data that provided a more coherent picture of program operations and institutional capacity. These approaches also demonstrate how existing centralized systems and structured data at RCMIs can improve the visibility of an RCMI’s research productivity and contributions to institutional research capacity.

##### Using Mixed-Methods and Narrative Approaches to Deepen Evaluation Insights

Mixed-methods approaches that included non-quantitative data generated richer, more contextualized findings. One evaluator commented that “We collect qualitative data through interviews to capture contextual factors such as research climate.” Surveys, interviews, open-ended questions, and success story interviews were used to capture investigator experiences, aspects of the research environment, and program impacts. The combination of structured scales and qualitative reflections allowed evaluators to better understand the mechanisms behind quantitative outcome metrics, document the lived experiences of investigators and community partners, and better understand the implementation context. The triangulation of non-quantitative and quantitative data also deepened evaluators’ understanding of program outcomes—i.e., how an RCMI supports advances in research careers, enhanced institutional capacity, and positive community impacts.

##### Systematic Tracking of Research Productivity and Collaboration Patterns

Evaluators provided several examples that demonstrate how systematic tracking using non-quantitative approaches coupled with traditional mixed methods has expanded their ability to document research productivity and collaboration dynamics. Coding publication databases, categorizing grant submissions, longitudinal tracking of proposal and manuscript development, and documenting pilot project and policy-related outcomes enabled sites to better characterize research foci and productivity. At one site, SNA enhanced its understanding of collaboration patterns by visualizing authorship networks and partnership trends, which provided a more comprehensive view of interdisciplinary, collaborative engagements. The use of reflection surveys by one site also strengthened its understanding of collaboration dynamics.

##### Documenting Community-Engaged Collaborations

CECs, as key components of current RCMIs, effectively documented partnerships, capacity-building activities, and relationship development using retrospective assessments and mixed-methods tools. These approaches captured the depth and evolution of community-engaged work, highlighting how partnerships strengthened over time and how institutional support contributed to community capacity. The ability to document these outcomes using non-quantitative methods was a notable success, particularly given the relational and context-dependent nature of community engagement. For example, including collaboration metrics in REDCap and annual reports has improved consistency and visibility of RCMIs’ community impacts.

##### Data-Driven Program Improvements

Open-ended items in surveys, interviews, and narrative tools provided actionable insights that helped programs identify challenges, refine activities, and implement targeted changes. Success stories and reflections surveys offered evaluators windows into barriers encountered by investigators and the solutions that emerged. One evaluator noted that “Findings are used to directly inform program changes.” These data-driven adjustments were believed to enhance program responsiveness (e.g., continuous quality improvements) and contribute to more effective support for researchers and community partners.

##### Structured Use of IDPs and Mentorship Evaluations to Improve Research Readiness and Competitiveness

RCMIs place special emphasis on preparing early-stage investigators to be competitive in the NIH grant process and to achieve career advancements. The systematic use of IDPs, mentorship assessments, and incorporating items for narrative into related surveys represented a significant advancement in tracking early investigator development and mentoring quality. These tools provided structured insight into professional growth, institutional barriers, and the effectiveness of mentoring relationships. By integrating IDPs and mentorship evaluations into routine practice, programs strengthened their ability to monitor career trajectories and support investigators in achieving research readiness and competitiveness. One evaluator commented that “PPP applicant surveys capture institutional barriers and support that shape professional growth.”

#### 3.2.2. Challenges

Sites described notable challenges in utilizing non-quantitative approaches to evaluation (Table 6). Specific findings for each theme are presented after the table.

**Table 6:** Summary of themes identified as “challenges” to implementing non-quantitative approaches to RCMI evaluations.

Challenges (Themes)	Examples
*Limited Capacity and Resources for Non-Quantitative and Traditional Qualitative Methods*	Insufficient training, staffing, and time; labor-intensive strategies; extensive data cleaning and interpretation needs
*Low Engagement and Participation*	Low survey response rates; inconsistent participation in tracking systems; low response to evaluation tools to assess mentorship and IDPs
*Misalignment and Inconsistency in Data Collection*	Variation across RP, PPP, and supplements; dispersed sources of collaboration data; lack of standardized indicators for collaborations and partnerships
*Technical and Infrastructure Limitations*	Limited access to utilization data; lack of specialized expertise required for some strategies; lack of centralized systems from which to review contextual data
*Timing and Recruitment Challenges*	Timing of data collection misaligned with reporting cycles; difficulty identifying participants post-program; need for early planning and integration of evaluation

##### Limited Capacity and Resources for Qualitative and Non-Quantitative Methods

A persistent challenge in RCMI evaluations was insufficient training, staffing, and time to support non-quantitative or non-traditional qualitative data collection and analysis. Some evaluators described minimal prior experience with non-quantitative methods and noted that large volumes of narrative data required substantial effort to manage within tight deadlines. One evaluator emphasized that “large volumes of qualitative data must be managed and analyzed within tight reporting timelines.” Another explained that “I have pretty minimal training or experience with non-quantitative methods.” Sites also noted that although non-quantitative and qualitative findings are valuable, “collecting and contextualizing [the findings] requires substantial effort,” underscoring the resource-intensive nature of collecting and reporting on narratives, document reviews, reflection surveys, success story interviews, and other non-quantitative data. Conducting an SNA or other nuanced approaches also requires extensive conceptualization, interpretation, and technical expertise that not all sites can sustain. These constraints made it difficult for evaluators to balance human and fiscal resources for methods that are complementary but not always central to core evaluation activities.

##### Low Engagement and Participation in Surveys, Tracking Systems, and Mentorship Tools

Low response rates and inconsistent participation across surveys, tracking systems, and mentorship evaluations posed significant barriers to data completeness. For example, annual surveys often achieved only modest participation, with one site reporting that “response rates are always rather low (50–60%)”. Other evaluators highlighted other challenges, noting that “the main challenge remains promoting timely data entry and sustaining engagement in completing surveys.” These participation gaps extended to mentorship evaluations and IDPs, where “variable participation among mentors and mentees limits follow-up on identified training needs,” reducing the reliable collection of data to document the effectiveness of these RCMI components. Further, these challenges limit evaluators’ ability to track early investigators’ developmental progress, and broader investigator development is not consistently monitored or tracked. As one evaluator noted, “Professional growth tracking of other [non-early-stage] RCMI investigators has not been a priority.”

##### Misalignment and Inconsistency in Data Collection Across Programs, Collaborations, and Community Partnerships

RCMI sites reflect substantial variation in how partnerships, collaborations, and program activities are structured and funded (e.g., through RP, PPP, and administrative supplements), making standardized data collection difficult. As one evaluator noted, “Given the breadth of how each RP, PPP, and supplement might engage partners, uniform data collection is not possible.” Also, collaboration data were described as dispersed across RCMI cores, with scientific collaborations receiving less attention (because they may be housed in multiple cores) than community partnerships (which are more formally embedded in the required CEC). Thus, RCMIs are more likely to have systematic strategies for documenting community partnerships than for documenting scientific collaborations. Other misalignments occur due to differences between the end of grant periods and the timing of a funder’s required reporting, or time lapses between when activities or events occur and when data are collected. One evaluator noted that “Funders may require early reporting…whereas the interview is designed to capture the full year”, and another indicated that “Annual surveys occur long after services were provided, reducing recall accuracy.”

##### Technical and Infrastructure Limitations, Including SNA and Utilization Data

Several sites faced technical barriers related to data systems, analytic tools, and access to utilization data. SNA, while valuable, required extensive data cleaning and specialized expertise that was not consistently available. The site that used SNA reported hiring “a graduate student for a whole semester just to clean the SNA data” and that “the expertise in SNA may not be available at all RCMI sites.” Some evaluators described limited access to laboratory and equipment utilization data managed by cores, which could provide more in-depth characterization of users and the facilities’ impact on institutional research capacity. These infrastructure gaps hindered the ability to conduct comprehensive and integrated assessments. Combined with limited access to centralized data systems, these time and resource demands contributed to the difficulty of implementing technically complex, non-quantitative strategies.

##### Timing and Recruitment Challenges for Post-Program Evaluation or Follow-Up

The misalignment between reporting cycles and program activities created additional challenges. As noted earlier, one evaluator explained, “funders may require early reporting… whereas the interview is designed to capture the full year”; and annual surveys administered long after services were delivered suffered from reduced recall accuracy. Recruitment for post-program non-quantitative activities was similarly difficult. Sites emphasized the need for early planning, setting expectations for RCMI-affiliated participants to respond to requests for follow-up evaluation activities, and setting clear criteria for recruiting participants for post-programs.

### 3.3. Recommendations for the Expansion of the Primary and Secondary Targets (Guiding Question 3)

The final guiding question asked respondents, “are there any missing primary or secondary targets that should be added (building on the 2020 [5] paper)? What qualitative (non-quantitative) evaluation outcomes would you recommend for each new item you proposed?” The original primary and secondary targets identified by Sy et al. [5] serve primarily as broader evaluation domains rather than as specific, measurable indicators. Respondents recommended that while the original four primary targets remain foundational, they must be expanded and qualitatively operationalized to better capture institutional transformation, equitable research environments, and longitudinal societal impact.

#### 3.3.1. Identification of New Primary Targets and Qualitative/Non-Quantitative Metrics

As a result of the thematic synthesis of 18 data excerpts, we identified three distinct new primary targets that address critical gaps in the original framework regarding equity and sustainability. First, the proposed target “Foster an Equitable and Responsive Research Environment” evaluates institutional health by documenting procedural changes made in response to investigator well-being and equity feedback. The recommended non-quantitative outcome involves key informant interviews with department chairs, deans, and program directors to capture specific examples of internal policy or resource allocation changes made directly in response to feedback regarding burnout or research equity. Second, the proposed target “High-Quality, Reciprocal, and Equitable Collaborations” shifts the focus from a mere count of partnerships to the quality and integrity of the process. This is documented through in-depth project lead interviews focusing on narrative accounts of challenge resolution, mutual resource exchange, and empirical evidence of equitable power distribution in decision-making. Finally, the proposed target “Achieving Sustainable Community and Policy Impact” measures longitudinal societal benefits using semi-structured interviews with Community Advisory Board (CAB) members and investigator surveys; tracks research disseminated or co-authored by community partners and policy changes at the institutional, local, state, federal, and clinical practice levels; and documents the community impact resulting from policy changes. These approaches identify tangible, ongoing shifts in community health knowledge and regional policy that result from research partnerships, providing evidence of true capacity transfer and adoption.

#### 3.3.2. Qualitative Refinement of Existing Secondary Targets

Beyond the creation of new primary targets, the data highlight a methodological deepening of established secondary targets. For grants and peer-reviewed publications, the findings suggest a shift toward longitudinal tracking systems that utilize standardized coding variables to classify records. One approach identified in the data involves adding a comprehensive set of approximately 15 distinct coding items to each record, enabling evaluators to track critical variables such as early-stage investigator (ESI) and underrepresented (UR) status at the time of submission. These granular data are vital for documenting institutional firsts, such as instances where Native American Investigators obtained their first NIH K-series (Career Development Program) awards in their institution’s history, serving as non-quantitative proof of the RCMI program’s impact on diversifying the biomedical research workforce.

Furthermore, the secondary target of scientific dissemination has evolved to include content analysis of real-world impact. By utilizing bibliometric and translational measures, such as Altmetric Explorer, evaluators can qualitatively assess how research products are integrated into clinical guidelines, policy documents, and mainstream media. This approach allows an evaluation team to document early attention and public engagement well before traditional citation counts emerge. Similarly, for the primary target of professional growth, refinements in the secondary targets include the collection of mentor–mentee paired narratives. These accounts allow both parties to reflect on specific challenges, focusing on how mentors adapted their approach to cultural humility and how mentees developed self-efficacy and independent problem-solving skills.

The following themes represent the synthesis of the 18 qualitative excerpts collected from the multi-site evaluation teams (Table 7).

##### Conceptual Map of Foundational and Expanded Domains to Inform RCMI Evaluation

**Figure 1:**
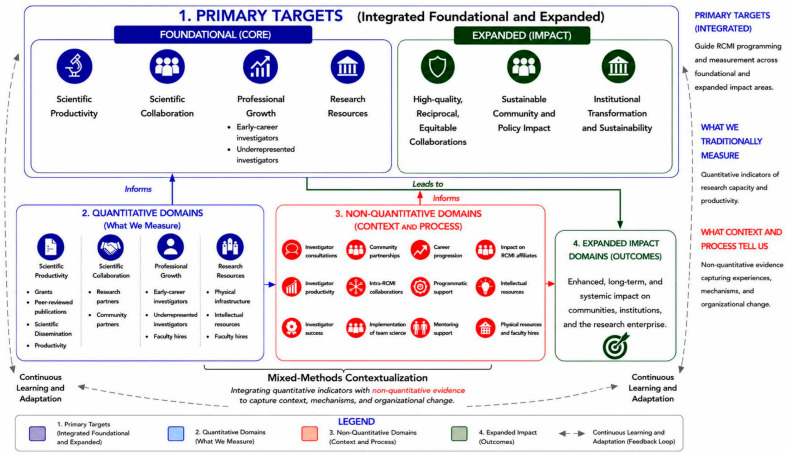
RCMI evaluation conceptual map: foundational and expanded domains.

By leveraging integrated data systems across RCB programs like the RCMI, evaluators can transform individual “success stories” into empirical evidence of systemic change. Based on our findings, we propose an expanded RCMI evaluation conceptual map that offers a set of quantitative [5] and non-quantitative domains that can be used to identify key evaluation questions across the consortium to move from site-specific to collective consortium-wide evaluation. Our findings indicate that both quantitative and non-quantitative approaches are important for conveying evaluation results, as they deepen interpretation, provide context, and offer insights about lived experience, enriching evaluators’ ability to convey individual, institutional, and community impacts that are critical for an RCB program like the RCMI. Gallo et al. underscore the importance of mixed methods for deepening our understanding of disparities and identifying solutions to improve health [44]. This integrated framework (Figure 1) visualizes the strategic progression of RCMI evaluation. The grey and blue layers (1 and 2) capture foundational, traditional quantitative metrics such as grants and publications. The orange layer (3) adds critical non-quantitative context and process (interviews and narratives). The green layer (4) illustrates the ultimate goal: sustainable systemic change in community health and policy. The dynamic broken arrows indicate the continuous feedback loops for iterative learning and adaptation. This tiered structure demonstrates that both numerical metrics and narrative impact must be paired to comprehensively measure research capacity and community transformation.

## 4. Discussion

### 4.1. Approaches to Advance RCMI Consortium-Based Evaluation

This reflective practice-based multi-site study identified actionable approaches to elucidate *how* RCMIs build investigator and institutional capacity, thereby addressing the utility of evaluation findings for guiding programmatic change [6]. When evaluators employ Developmental Evaluation and Responsive Feedback frameworks drawing on non-quantitative and mixed-method approaches, they enrich our contextual understanding for decision-making to inform change [20,21]. Non-quantitative strategies inform all stages of a comprehensive evaluation, including formative or process (program implementation/fidelity), outcome (changes that result from the strategies employed by RCMIs), and impact (long-term community and or societal change resulting from the RCMI program). Drawing on Emmons et al., the expansion of the TSBM can deepen our understanding of RCMI outcomes related to the implementation process [15]. With this in mind, the non-quantitative evaluation approaches described in this paper allow for understanding *how* and in *what contexts* impact happens [15]. Consistently, throughout all primary targets, sites used strategies to identify and understand successes and challenges and inform programming beyond formative questions for timely adaptations [20]. The following findings illuminate non-quantitative approaches for contextualizing the implementation of RCMI programs and understanding their impact on institutional capacity.

Evaluation domains related to *scientific productivity* (e.g., consultations, contextual factors) help explain *how* and *why* productivity happens (or why an investigator may not be productive). Non-quantitative evaluation domains related to *scientific collaboration* show how partnerships are formed and strengthened and how they yield coequal benefits. These approaches inform how interdisciplinary collaborations with investigators at various career stages emerge within RCMI sites, and how investigators expand their networks and capacity for team science. A robust, multi-method strategy for understanding both the processes and outcomes of scientific collaboration is vital in contexts where community engagement and disparity-oriented research are central [10]. These strategies should include measures of internal and external validity to ensure that programs and policies are sustainable in clinical and community settings [19].

Domains to assess *professional growth* have implications for evaluation practice, including adopting a mixed-methods, longitudinal, and multi-domain approach that includes measures of career trajectory, barriers/facilitators, mentoring quality, benchmarks for scholarly deliverables, investigator perceptions of programmatic support, and narrative reflections to drive continuous program improvement, document investigator-level capacity building, and demonstrate return on investment. RCMI non-quantitative evaluation strategies designed to assess professional growth could inform TSBM by deepening its understanding of workforce development focused on translational researchers [13,15].

The evaluation domains related to *research resources* aligned directly with the secondary targets identified by Sy et al. [5] (intellectual resources, physical resources, and faculty hires). Our findings in this domain focus on approaches that systematically document changes in capacity, quality, and resource use across the intellectual, physical, and human capital domains, drawing on qualitative evidence. Structured monitoring tools should be included to assess how research resources, e.g., support, infrastructure, and faculty recruitment, translate into improved research rigor, efficiency, and sustainability.

### 4.2. Non-Quantitative Evaluation Successes and Challenges

Across RCMI sites and primary targets, evaluators shared resources and structural challenges, including limited capacity for traditional qualitative or non-quantitative research, dispersed sources of collaboration data, and institutional barriers to tracking resources and impacts over time. Yet the collective findings also highlight substantial innovation and progress in the implementation of non-quantitative approaches. Together with the traditional mixed-methods approach, non-quantitative methods are becoming a defining strength of RCMI evaluations. For example, to contextualize RCMI progress and impacts, evaluators increasingly pair quantitative metrics with non-quantitative data collected through approaches such as social network analysis, reviews of project descriptions, content analysis of the focus of researchers’ publications, and narratives from success story interviews and reflection surveys. Also, secondary data systems, such as institutional grant databases with project descriptions, publication repositories that can be coded to align with RCMI goals, and REDCap systems that can pair quantitative and non-quantitative data, play critical roles in reducing burden and improving data integrity. Our findings align with Zhang et al. [25], who recommend mixed methods integrating quantitative and qualitative data for RCB evaluations.

However, non-quantitative approaches to analyzing the data in these repositories need further development. For instance, can evaluators use these repositories to content-analyze these data to (1) characterize the topics and nature of the research published or grants submitted and awarded and (2) determine the extent to which these characterizations align with the goals of the respective RCMI or the overall RCMI initiative? Use of non-quantitative methods to assess the primary targets is evolving, offering promising non-quantitative avenues for capturing enriched information about the primary targets, especially for documenting the outcomes of an entire research consortium. However, these approaches require early planning, dedicated resources, and institutional support to be fully effective.

Challenges and constraints, such as lack of qualitative/non-quantitative expertise, limited staffing or consulting resources dedicated to evaluation, inconsistent data collection and tracking systems, low participant engagement, and misaligned reporting cycles, reflect structural or system-level conditions rather than isolated program issues. Therefore, shifts in structural and system-level policies are needed to ensure more successes with non-quantitative approaches incorporated into evaluations implemented within varied RCMI contexts. A grant requirement for a specific evaluation budget or inclusion of dedicated evaluation staffing in an RCMI could address some of these challenges. However, funding is also needed for interoperable data systems that reduce duplication and manual data cleaning and improve participant tracking. Although the lack of standardized indicators across RP, PPP, and supplements is being addressed by the establishment of common data elements (CDEs) across RCMIs, these are largely quantitative. Policies are needed at the federal level to integrate non-quantitative and mixed-methods approaches into cross-RCMI evaluation planning. Alignment of reporting timelines with realistic data collection cycles is also warranted, and funding is needed to train RCMI evaluators in non-quantitative and mixed-method approaches. Together, these structural and system-level changes would move evaluation from a labor-intensive, fragmented activity at each RCMI to a coordinated, cross-institutional function capable of producing consistent, high-quality non-quantitative evidence that complements the primarily quantitative CDEs.

### 4.3. Expansion of the Primary Evaluation Targets

The proposed expansion of the primary targets focuses on the impact of RCMIs while underscoring the reciprocal benefits of research and the added value of RCMI research to communities. The proposed targets include *Foster an Equitable and Responsive Research Environment; High-Quality, Reciprocal*, *and Equitable Collaborations*; and *Achieving Sustainable Community and Policy Impact.* The results underscore a strategic trend toward the use of impact narratives and the alignment of evaluation instruments with the TSBM, specifically in the community and policy domains [14]. This integration facilitates a comprehensive description of research impact across scientific, clinical, and policy domains. Furthermore, the use of innovative qualitative tools, such as Photovoice to evaluate identity and belonging within specialized laboratory environments, demonstrates the program’s evolution toward capturing the human element of scientific success [41]. Collectively, these data-driven findings argue for an evaluation model that treats scientific achievement as a multifaceted narrative of institutional and community transformation.

### 4.4. Integrating Theory and Reflective Methods to Inform Evaluation Practice

This meta-evaluation advances evaluation theory, science, and practice by demonstrating how reflective, cross-site learning can identify the contextual mechanisms that traditional metrics overlook. By identifying non-quantitative domains that explain *how* research capacity is built, not just *what* outputs or outcomes are produced, this work operationalizes core principles from meta-evaluation (continuous quality improvement), implementation science (attention to context, readiness, and mechanisms of change), and mixed-methods inquiry (using qualitative evidence to explain quantitative patterns). The multi-site reflections align with the core principles of meta-evaluation [47,49]. For example, challenges such as inconsistent data capture, limited non-quantitative and qualitative research capacity, and fragmented infrastructure mirror the types of systemic constraints that meta-evaluation theory predicts will undermine the credibility and usefulness of evaluation systems. The successes reinforce the recent literature that demonstrates how reflective practices can strengthen evaluation design, enhance data quality, and support continuous improvement [4]. The emergent non-quantitative domains also map closely onto implementation science frameworks. For example, evaluators’ descriptions of contextual barriers, institutional readiness, and the influence of settings on RCB reflect CFIR’s [55] emphasis on understanding the multilevel determinants of implementation success. The use of longitudinal tracking systems, reflection surveys, and community-engaged methods aligns with RE-AIM’s focus on documenting how RCMI activities are taken up by investigators, cores, and community partners and sustained over time [19]. Further, this study’s emphasis on pairing non-quantitative data with traditional metrics reflects the logic of mixed-methods evaluation. Approaches such as success stories, social network analysis, and document reviews exemplify approaches that mixed-methods scholars describe as “explanatory” or “complementary” [42], as found in studies that use qualitative data to illuminate the processes underlying quantitative indicators [43]. For RCB initiatives, where the pathways to returns on investment (ROI) are relational and thus cannot be fully captured solely by numeric indicators, this is particularly useful. Gallo et al. [44] argue that mixed methods are indispensable for understanding health equity and translational science outcomes. However, the present findings extend this argument by proposing new domains for theory-driven evaluations of processes and outcomes focused on assessing the effectiveness of RCB for underrepresented researchers and within institutions and communities with complex histories. Thus, by grounding non-quantitative approaches in established evaluation and implementation frameworks, RCMI evaluators propose a more coherent, theory-informed model for RCB evaluation that captures institutional transformation, investigator development, and community impact in ways that existing quantitative CDEs and common metrics cannot. Moreover, this theoretical integration strengthens the argument posited by investigators using the TSBM [25] and other models that non-quantitative approaches are not ancillary but essential for capturing the mechanisms, contexts, and relational dynamics through which effective RCB occurs in complex settings [43,44].

### 4.5. Study Implications

Future work should include the expansion of our RCMI evaluation model (Figure 1) to encompass the TSBM domains. The authors suggest a multi-site mixed-methods study to operationalize RCMI-relevant impact-oriented questions for each domain in the TSBM with both primary and secondary data to ensure methodological rigor.

This reflective practice-based study reveals several implications for strengthening the RCMI consortium-wide evaluation:

1. Standardization is essential.
  Shared definitions for ROI, CDEs, and collaboration metrics would improve comparability and reduce burden. Although the types of non-quantitative information collected tended to cluster around similar themes (as shown in Table 1, Table 2, Table 3 and Table 4), it is noteworthy that evaluators utilized several different methods to collect information within each respective domain. For example, five different approaches (milestone reviews, longitudinal tracking, collaborative assessment, IDP reviews of progress, and surveys) were implemented to measure various aspects of career progression. It is unclear whether the data collected via such varying methodologies are comparable. To provide a report on the overall impact of the RCMI network, standardized approaches to non-quantitative evaluation should be adopted.
2. Qualitative capacity must be expanded.
  Training, templates, and analytic tools are needed to support evaluators with limited non-quantitative experience.
3. Mixed-methods approaches should be institutionalized.
  Embedding open-ended items, reflection surveys, and success story frameworks into routine data collection could enhance our understanding of program impact.
4. Centralized data systems improve sustainability planning.
  REDCap, publication databases, and grant management systems reduce burden and strengthen data quality. However, non-quantitative approaches are needed to fully understand the qualitative contents of entries in these repositories and to further illuminate RCMIs’ impact.
5. Professional growth evaluation should extend beyond pilot investigators.
  Systematic tracking of career progression and the research climate would provide a fuller picture of capacity building for junior and senior investigators.
6. Resource evaluation requires institutional commitment.
  Sustainable evaluation models depend on alignment between evaluation teams, core leadership, and institutional administration.

Collectively, these findings underscore the importance of and provide directions to RCMI consortium evaluation on using coordinated, mixed-methods, and capacity-building approaches [32]. As RCMI sites continue to document their research impacts, these insights can guide more robust and meaningful non-quantitative evaluation strategies that more holistically capture the full impact of RCMI investments on investigators, institutions, and communities.

### 4.6. Limitations

Eight individuals representing nine RCMI sites participated in this reflective multi-site study. Because these evaluators were recruited from an evaluation-focused CoP, it is likely that they have a strong interest in and knowledge of evaluation practices, including non-quantitative approaches, and may encounter relatively few evaluation challenges. The purpose was to obtain rich descriptive results, not to be representative of RCMI evaluation practices or challenges in general. The convenience sample of CoP members only should be interpreted as practice-informed evaluative reflections intended to advance methodological learning within consortium-based research capacity-building contexts. As such, the purpose was not to generalize across the broader RCMI consortium.

The use of a shared Google document for response collection was intended to support reflection and idea generation (similar to a focus group), but it may have introduced potential contamination of responses and conformity bias. Future meta-evaluations might consider collecting information from respondents separately to mitigate this risk. We acknowledge that using asynchronous written responses in a shared Google document did not provide opportunities for probing or standardization of response depth or richness. Because this is not a qualitative research study, we do not recognize this as a methodological limitation per se. We did not aim for methodological triangulation. This study primarily relied on written evaluator reflections as the principal data source; confirmability was strengthened through prolonged engagement during the data collection process, iterative reflection among CoP members, collaborative review of responses, consensus-building during thematic interpretation, and maintenance of audit trails documenting analytical decisions and revisions throughout the project.

Several responses provided by evaluators were excluded from thematic analyses because they were non-specific, quantitative in nature, or vague in their descriptions of non-quantitative evaluation questions and, therefore, limited in their interpretive value. Future meta-evaluations should incorporate a pilot testing phase that engages non-participants to validate the reflective questions and ensure that they effectively elicit the desired types of responses.

Another limitation was posed by the use of generative AI. Because generative AI can introduce errors or surface excerpts without full contextual awareness, all selected quotes were manually checked by two researchers, who examined each quote in the context of the original narratives. The interpretive authority remained with the research team, and only AI-selected text passages/verbatim quotes that were accurate and contextually fit were used in the analysis. Finally, this article is based on a “post hoc” analysis of RCMI evaluator experiences; it was not initiated with a theory to be tested. This study was deliberately designed using a convenience sample and a clear need (identified in the CoP) to leverage strengths from RCMI evaluation practice to fill gaps in the RCMI evaluation model.

## 5. Conclusions

The authors recommend an approach to drawing on both the quantitative targets outlined in Sy et al. 2020 [5] across RCMI sites in the consortium and non-quantitative strategies, such as those identified in this project, to provide context, communicate evidence of success, and inform programmatic changes to enrich the findings and strengthen the rigor of the RCMI evaluation practices. Standardizing processes for integrating evaluation data and information across multiple sources can help to communicate impact and strengthen the collective ability of sites to tell the story of the RCMI consortium.

## Figures and Tables

**Table 1:** Scientific productivity: non-quantitative evaluation domains and approaches.

Non-Quantitative RCMI Evaluation Domains	Approaches
Investigator productivity (n = 3) **	
Scholarly products resulting from community research studios	Vignette (individual example)
Descriptions of community/professional conference dissemination efforts, honors/awards, and promotions	Productivity reports
Co-developed core milestones that guide and contextualize productivity goals	REDCap (research electronic data capture) for formative, progress, post-assessment, and exit surveys
Investigator consultations (n = 2) **	
Grant preparation activities such as consultations, development, resubmissions, and reviewer feedback	Longitudinal tracking
Descriptions of reasons for the studio consultations (advice/guidance on any research related issue)	Vignette (individual example)
Investigator success (n = 2) *	
Profiles of “productive” Pilot Program Project (PPP) investigators describing their subsequent productivity/success	Profiles (individual example)
PPP submissions/outcomes, dissemination activities that generate preliminary data, and increased research visibility	Secondary data from Community Engagement Core (CEC)

* *n* = number of text excerpts relating to each theme; ** one response was split and reported under two themes as it related to both.

**Table 2:** Scientific collaborations: non-quantitative evaluation domains and approaches.

Non-Quantitative RCMI Evaluation Domains	Approaches
Community partnerships (n = 13) *	
Types of external partners working across the RCMI (research projects), relationship building, nature of the collaboration, and strengthened relationships	● Longitudinal tracking ● Descriptions of organizations
CEC Community Campus Partnership Support (CCPS) feedback to improve the program; how did participation build capacity to engage in health disparities research with community/university partners?	● Retrospective pre/post-survey
CEC Community Engagement Studio (CES) community expert-driven changes to the research project that resulted; new community partnerships; strengthened partnerships	● Vanderbilt CES survey [52] ● CES outcome survey ● Activity/consultation logs
Did the RCMI strengthen investigators’ ability to develop and sustain relationships with community partners?	● Investigator Reflection Survey
Did Community Expert Board (CEB) participation benefit CEB members’ organizations or the populations they serve?	● CEB reflection survey
Intra-RCMI Collaborations (n = 6) *	
Inter-core collaboration an indicator of institutional research capacity	● Administrative core monitoring ● Goal-oriented milestone tracking
Expansion of scientific collaborations among faculty across career stages; pilot project collaborations	● Social Network Analysis (SNA)
Collaborations that emerge/what facilitates them/barriers encountered; shared activities, co-developed outputs from interdisciplinary and community–academic partnerships	● Annual progress report ● Milestone tracking ● Interviews (semi-structured)
Implementation of team science (n = 5) *	
Whether/how the RCMI expanded investigators’ network of health disparities research collaborators; how has the RCMI impacted your ability to successfully engage in team science?	● Investigator Reflection Survey
Collaborative networks, assessed diversity, and connectivity among investigators, disciplines, and institutions; jointly developed proposals, co-authored manuscripts, and new teams	● Social Network Analysis (SNA) ● Tracking/center feedback form ● Systematic data capture

* *n* = number of text excerpts relating to each theme.

**Table 3:** Professional growth: non-quantitative evaluation domains and approaches.

Non-Quantitative RCMI Evaluation Domains	Approaches
Career progression (n = 9) *	
Identify barriers, facilitators, and opportunities for targeted interventions	● Milestone reviews
Document and monitor promotion and tenure changes among RCMI-affiliated faculty/postdocs, awards, student trainee outcomes—current position	● Curriculum Vitae (CV)/milestone reviews ● Longitudinal participation tracking ● Collaborative assessment
Grant/publication preparation (consultations, draft development, resubmissions, and reviewer feedback)	● Longitudinal tracking
Assess goal attainment, identify support needs, and guide mentoring discussions	● Individual Development Plan (IDP) review of progress ● Formative/progress/post-surveys
Programmatic supports (n = 9) *	
Interest in professional development opportunities; identify needs/assess perceptions of available support	● Interviews (semi-structured)
Open-ended questions to gain insights into the PPP application process and scientific review/feedback	● PPP applicant survey
What resources were available to accomplish goals/activities? What activities resulted from the use of resources? What are the immediate results of these activities?	● Key Persons’ Survey
Revisiting professional growth goals to ensure alignment with the RCMI’s mission and goals	● Milestone reviews ● Complementary data sources
Mentoring supports (n = 8) *	
Satisfaction of PPP leaders with various center-sponsored professional development activities	● Satisfaction survey
Contextual factors: challenges/solutions; facilitators of progress and success; factors that influenced/impeded progress; suggestions for improvement	● Open-ended questions ● Formative interviews
Community Engagement Studio (CES) outcomes: changes in investigators’ perspectives about community feedback or changes to study design/dissemination plans	● Community expert engagement
Factors that helped PPP investigators participate in individualized mentoring; self-reported skill enhancement; changes in self-efficacy/career readiness	● Formative interviews
Mentor accessibility, feedback, support for independence, contributions to scholarly productivity	● Validated mentorship evaluation tool [53]
Describe whether the program strengthened investigators’ ability to mentor early-stage investigators (ESIs)	● Survey with open-ended questions
Impact of RCMI on affiliates (n = 4) *	
Contextualize return on investment (ROI) calculation with success stories	● Multiple evaluation data sources
Impact of the RCMI on affiliated faculty, staff, students, recharge users, community partners, and volunteers; ways (if any) in which affiliates’ involvement in the RCMI impacted their career; impact of collaborative efforts; impact on institutional capacity	● Survey (open-ended questions) ● CDC Success Stories Template [54] ● Survey (RCMI affiliates)

* *n* = number of text excerpts relating to each theme.

**Table 4:** Research resources: non-quantitative domains and approaches.

Non-Quantitative RCMI Evaluation Domains	Approaches
Intellectual resources (n = 7) *	
Awareness/use of available support and resources to advance research skills	● Interviews
Changes (individual/researcher capacity) because of RCMI activities (grounded by inner settings based on attributes from implementation science)	● Content analysis using the logic model components as headers
How RCC consultations (study design, statistical methods, data analysis, grant development) lead to improved proposals, manuscripts, and analytic rigor	● Systematic data capture
Applicants identify institutional barriers and support for writing PPP grant proposals	● PPP applicant survey
Identify barriers and assets to individual and institutional research readiness and inform RCMI programming	● Research readiness survey
Development of new research methodologies, consultations, and training in analytic techniques	● Systematic data capture
Physical resources (n = 5) *	
Changes (research infrastructure enhancements) because RCMI activities are grounded by outer settings based on attributes from implementation science	● Content analysis using logic model components as headers
Resources available, support needs, expanded analytic capabilities, gaps in equipment or infrastructure	● Formative/post-surveys
Monitoring progress on equipment acquisition, laboratory improvements, and other resource expansion efforts	● Infrastructure-related milestones
Faculty hires (n = 2) *	
Recruitment core retention of new investigators hired	● Goal-oriented productivity milestones
Feedback from investigators about new resources and improved research efficiency resulting from faculty hires	● Documented feedback

* *n* = number of text excerpts relating to each theme.

**Table 7:** Synthesis of findings: opportunities to expand RCMI evaluation practice.

Theme	Key Findings and Recommendations
Structural Evolution	Observed that 2020 targets currently serve as broad domains rather than specific metrics; recommended alignment with RCMI Coordinating Center efforts to operationalize variables consortium-wide.
New Primary Target: Equitable Collaborations	Proposed new target: *High-Quality, Reciprocal, and Equitable Collaborations*. Recommended in-depth interviews with leads to document power distribution and mutual resource exchange.
New Primary Target: Sustainable Impact	Proposed new target: *Sustainable Community and Policy Impact.* Recommended semi-structured interviews with CAB members to identify longitudinal shifts in practice and policy, documenting policy changes and community-level indicators (e.g., improved population health) resulting from RCMI-supported work, and community co-authorship on RCMI products.
Refinement of Secondary Targets	Emphasized robust longitudinal tracking for *grants and publications*. Identified the use of approximately 15 distinct coding items to track ESI/UR status, core engagement, and “institutional firsts” (e.g., NIH K99/R00 awards).

## Data Availability

The data supporting the results and conclusions of this article will be made available by the authors on request. Data were provided by individual evaluators without prior review by other staff or administrators from their institutions, with the understanding that no identifiable information would be made available to the public and that the data would be protected from view unless expressly approved by the respondent or their institution. Because we only received responses from nine of the 23 RCMIs, this reduces the likelihood that we can protect individual institutions from instances in which excerpts in the qualitative data might be associated with a particular institution.

## Supplementary Materials

The following supporting information can be downloaded at: https://www.mdpi.com/article/10.3390/ijerph23060747/s1, File S1: Guiding Questions for RCMI Evaluator Reflection.

## Abbreviations

The following abbreviations are used in this manuscript:

AI	Artificial Intelligence
CAB	Community Advisory Board (for an RCMI CEC)
CCPS	Community Campus Partnership Support (of an RCMI CEC)
CDC	Centers for Disease Control and Prevention
CDEs	Common Data Elements (set of metrics collected across programs)
CEB	Community Expert Board (of an RCMI CEC)
CEC	Community Engagement Core (of an RCMI)
CES	Community Engagement Studio (of an RCMI CEC)
CFIR	Consolidated Framework for Implementation Research
CoP	Community of Practice
CTR	Clinical and Translational Research
CTSA	Clinical and Translational Science Award (NIH grant program)
CV	Curriculum Vitae
ESI	Early-Stage Investigator (NIH designation for researcher status based on degree or clinical training completed within no more than 10 years)
IDP	Individual Development Plan (individualized mentoring plan in RCMIs)
K-series	NIH Awards for Career Development Programs (e.g., K-01, K, 08, K-99, etc.)
K99/R00	NIH Pathway to Independence Award (mentored research with a K99 followed by independent research with an R00)
NCATS	National Center for Advancing Translational Sciences (at NIH)
NIH	National Institutes of Health
NIMHD	National Institute on Minority Health and Health Disparities (NIH)
PPP	Pilot Project Program (of an RCMI)
RCB	Research Capacity Building
RCC	Research Capacity Core (of an RCMI)
RCMIs	Research Centers in Minority Institutions (sites funded by NIMHD grants)
RCMI-CC	RCMI Coordinating Center (helps NIMHD and RCMI achieve collectively)
RE-AIM	Reach, Effectiveness, Adoption, Implementation, and Maintenance
REDCap	Research Electronic Data Capture (browser-based data collection system)
RePORTER	Research Portfolio Online Reporting Tools (NIH repository of grants)
RPs	Research Projects funded through the RCMI that receive support from all cores
ROI	Return on Investment (tangible and intangible benefits of an RCMI grant)
SNA	Social Network Analysis (research method to understand interactions)
TCCs	Transdisciplinary Collaborative Centers
TSBM	Translational Science Benefits Model (evaluation framework to demonstrate impact of research in real world)
UR	Underrepresented Researcher scholar
