# Supplementary material for: Contextualizing Evaluation in Research Consortia: A Reflective Case Study from the Research Centers in Minority Institutions (RCMIs) Program
Source: Int J Environ Res Public Health. 2026 Jun 2;23(6):747. doi: 10.3390/ijerph23060747 (PMC13299528; doi:10.3390/ijerph23060747)
Supplement: Supplementary file 1 [file ijerph-23-00747-s001.zip › ijerph-4260424-supplementary.pdf]

Guiding Questions for RCMI Evaluator Reflection

Purpose of this paper: In 2020, a group of RCMI evaluators co-authored an evaluation publication. Building on Sy et al. 2020 this follow-up manuscript will focus on key strategies for augmenting evaluation approaches to contextualize non-quantitative metrics such as success stories to document RCMI impact. Findings will be de-identified and shared in aggregate.

Goal of this request: Compile best practices from RCMI evaluators about RCMI evaluation using the guiding questions below.

Instructions:

1. Review the guiding questions below and share any best practices from your RCMI.
   - Do not feel like you have to provide a response for each item.
   - Feel free to review other folks' contributions to get your wheels spinning.
   - There are no wrong answers- please stay within the bounds of the questions posed below.
2. Keep your ideas in a paragraph or bullet form.

Guiding questions:
There are three primary questions below (I-III). We are asking folks to reflect on each question thinking about the 4 primary targets identified in the 2020 Sy et al. paper. See the table on the last page if you need some more context.

I. **What evaluation questions or lessons learned document/describe RCMI impact or outcomes beyond quantitative metrics and measures?**

   a. **Increase Scientific Productivity**

   b. **Increase Scientific Collaborations**

   c. **Foster Professional Growth**

   d. **Expand Research Resources**

II. **Share any challenges and/or successes related (directly) to the evaluation work you described in the first question (I) above (related to each primary target).**

   Please define success for your respective site based on the evaluation approaches you discussed above (**not** that results/findings from that evaluation work).

   a. **Increase Scientific Productivity**

   b. **Increase Scientific Collaborations**

c. **Foster Professional Growth**

d. **Expand Research Resources**

III. **Are there any missing primary or secondary targets that should be added (building on the 2020 paper)? What qualitative (non-quantitative) evaluation outcomes would you recommend for each new item you proposed?**

Here is a reference about the primary and secondary targets if needed (Sy et al. 2020)

Primary Targets	Secondary Targets
1. Increase Scientific Productivity	Grants<br>Peer-Reviewed Publications<br>Scientific dissemination<br>Community dissemination<br>Pilot project specific productivity
2. Increase Scientific Collaborations	Research partners<br>Community partners
3. Foster Professional Growth	Early Career Investigators<br>Underrepresented Investigators
4. Expand Research Resources	Physical infrastructure<br>Intellectual resources<br>Faculty hires*
